# Prevalence of frailty and its associated factors in older hospitalised patients in Vietnam

**DOI:** 10.1186/s12877-017-0609-y

**Published:** 2017-09-15

**Authors:** Huyen Thi Thanh Vu, Thanh Xuan Nguyen, Tu N. Nguyen, Anh Trung Nguyen, Robert Cumming, Sarah Hilmer, Thang Pham

**Affiliations:** 1 Department of Gerontology, Hanoi Medical University, Hanoi, Vietnam; 2 The National Geriatric Hospital, 01 Ton That Tung, Hanoi, Vietnam; 3 Sydney School of Public Health, University of Sydney, Sydney, NSW Australia; 4 Departments of Clinical Pharmacology and Aged Care, Royal North Shore Hospital and Kolling Institute of Medical Research, Sydney Medical School, University of Sydney, Sydney, NSW Australia

**Keywords:** Frailty, Older patients, Prevalence, Vietnam

## Abstract

**Background:**

Frailty is an emerging issue in geriatrics and gerontology. The prevalence of frailty is increasing as the population ages. Like many developing countries, Vietnam has a rapidly ageing population. However, there have been no studies about frailty in older people in Vietnam. This study aims to investigate the prevalence of frailty and its associated factors in older hospitalised patients at the National Geriatric Hospital in Hanoi, Vietnam.

**Methods:**

Prospective observational study in inpatients aged ≥60 years at the National Geriatric Hospital in Hanoi, Vietnam from 4/2015 to 10/2015. Frailty was assessed using the Reported Edmonton Frail Scale (REFS) and Fried frailty phenotype.

**Results:**

A total of 461 patients were recruited (56.8% female, mean age 76.2 ± 8.9 years). The prevalence of frailty was 31.9% according to the REFS. Using the Fried frailty criteria, the percentages of non-frail, pre-frail and frail participants were 24.5, 40.1 and 35.4%, respectively. Factors associated with frailty defined by REFS were age (OR 1.05 per year, 95% CI 1.03–1.08), poor reported nutritional status (OR 4.51, 95% CI 2.15–9.44), and not finishing high school (OR 2.18, 95% CI 1.37–3.46). Factors associated with frailty defined by the Fried frailty criteria included age (OR 1.07 per year, 95% CI 1.05–1.10), poor reported nutritional status (OR 2.96, 95%CI 1.43–6.11), not finishing high school (OR 1.58, 95% CI 1.01–2.46) and cardiovascular disease (OR 1.76, 95% CI 1.16–2.67).

**Conclusions:**

While further studies are needed to examine the impact of frailty on outcomes in Vietnam, the observed high prevalence of frailty in older inpatients is likely to have implications for health policy and planning for the ageing population in Vietnam.

## Background

Frailty is defined a state of increased vulnerability and decreased physiological reserve that can increase risk of poor outcomes in older adults [1]. The prevalence of frailty is increasing as the population ages [1, 2]. Over the past decades, research efforts have helped to provide a better definition and description of frailty, however there has been no gold standard for identifying frailty [3, 4]. Frailty is quite common in older hospitalised patients, with the prevalence ranged from 27 to 80% in [5–7]. There is increasing evidence that frailty can predict hospitalisation and adverse outcomes after discharge in older people, such as readmission, increased risks of disability and mortality [1, 8]. According to a systematic review and meta-analysis in 2016, frailty was significantly associated with higher hospitalisation risks (pooled Odds Ratio 1.90, 95% CI 1.74–2.07, *p* < 0.0001) [9]. Hospitalisation is a stressor event that can lead to a further deterioration and increase risk of dependence in older people, especially in the frail [7] [10]. In older hospitalised patients, frailty has been shown to have an influence on treatment strategies and responses to medications [11]. Understanding the prevalence of frailty in older inpatients may help improve care and prognosis [1].

The population in middle- and low-income countries are ageing rapidly. It is estimated that the speed of ageing in developing countries will outrun that of the developed countries in the near future [12]. However, there have been few published studies about frailty in developing countries. In a systematic review of frailty in developing countries up to the year 2014, there was only one study conducted in older hospitalised patients [13]. Vietnam is a typical developing country in Southeast Asia. In Vietnam, the population is ageing rapidly. In 2009, the percentage of people aged 60 or over was 8.7% and it is estimated that this figure will increase to 26.1% in 2049 [14]. However, there have been no studies about frailty in older people in Vietnam. Therefore, this study aims to examine the prevalence of frailty and its associated factors in older hospitalised patients at the National Geriatric Hospital in Hanoi, Vietnam.

## Methods

### Study population

This was a prospective observational study in older inpatients of the National Geriatric Hospital in Hanoi, Vietnam. Consecutive patients aged 60 years or older admitted to the hospital were recruited between April 2015 and October 2015. Exclusion criteria included severe illness (defined as dying or receiving intensive care), blind or deaf. Data was collected from medical records and included socio-demographics, detailed medical history, co-morbidities, clinical assessments and medication utilisation. All participants were interviewed for the Reported Edmonton Frail Scale (details are presented in Frailty definition), and for their nutritional status based on one question “How would you describe your diet” (with the response categories included “poor”, “stable” or “healthy”). For participants with severe cognitive impairments (7/461 participants), interviews were conducted with their caregivers. All participants were followed up for six months by conducting phone calls. This paper just reported the cross-sectional baseline data, the follow up data will be analysed and presented in a separate manuscript. The study was approved by the National Geriatric Hospital Ethics Committee and oral consent was obtained from all participants.

### Frailty definition

In this study, Fried frailty phenotype and the Reported Edmonton Frail Scale (REFS) were used to define frailty in all participants.

The Fried frailty phenotype included five criteria proposed by Fried with some adaptation in the slowness and low physical activity components. Participants who met at least three criteria were considered to be frail, whereas those with one or two criteria were pre-frail and those with no characteristics were defined as robust. The five criteria are as follows:

1. Unintentional weight loss of ≥5% or 4.5 kg in the last year.
2. Weakness: Grip strength was measured with a dynamometer (Jamar TM Hidraulic Hand Dynamometer 5030 J1 made in USA) and the value of the dominant hand was used. Weakness was defined by the lowest quintile of grip strength, adjusted for gender and body mass index (BMI) [15]. This method is consistent with the method in the original study conducted by Fried et al. in 2001 [16]. The cut-off points established were: (a) in men: BMI <18.50: grip strength <6.8 kg; BMI 18.50–24.99: grip strength <12.0 kg; BMI ≥25: grip strength <12.6 kg; (b): in women: BMI <18.50: grip strength <4.0 kg; BMI 18.50–24.99: grip strength <6.0 kg; BMI ≥25: grip strength <9.8 kg.
3. Low energy (Exhaustion): We used two questions from the Centre for Epidemiologic Studies Depression Scale (CES-D): In the last week “I felt that everything I did was an effort” and “I couldn’t get going”. Participants who answered “frequently” or “always” to at least one of these two questions were classified as having this criterion.
4. Slowness: We used the cut point of 5 s after walking 4 m to identify participants with slow walking speed [17, 18].
5. Low physical activity: Those subjects who answered “I rarely or never do any physical activities” were considered as having low physical activity.

All participants were also assessed with the REFS. This scale has been validated for use in acute inpatients in previous studies [19–24]. Compared to Fried’s frailty phenotype, the REFS is based on a questionnaire on how the patient functioned prior to the illness that brought them into hospital, is not heavily influenced by the acute illness, easy to apply for older inpatients and less time-consuming. This scale included nine frailty domains (general health status, functional independence, social support, medication utilisation, nutrition, mood, continence, functional performance, and cognition). The maximum score is 18, and the cut point used to identify frailty was 8, consistent with previous studies using this scale [19–24].

### Statistical analysis

Analysis of the data was performed using SPSS for Windows 20.0 (IBM Corp., Armonk, NY, USA). Categorical variables are presented as frequencies and percentages. Continuous variables are presented as means ± standard deviation. Comparisons between frail and non-frail participants were conducted with Chi-square test or Fisher’s exact test for categorical variables and Student’s t-test or Mann-Whitney test for continuous variables. Multivariate logistic regression was conducted to identify risk factors for prevalent frailty on admission. Univariate logistic regression was performed on all the potential risk factors for frailty: age, gender, nutritional status, underweight, comorbidities such as cardiovascular diseases (defined as having any of the following conditions: ischemic heart disease, peripheral vascular disease, aortic atherosclerosis, heart failure, stroke), hypertension, type 2 diabetes, chronic pulmonary diseases, renal impairment, cancer, osteoarthritis, anaemia and socio-economic factors (education, residential status). Variables that had a *p*-value <0.20 on univariate analysis were selected for multivariate analysis. A backward elimination method was applied and the final model retained variables significant at *p* < 0.05. All variables were examined for interaction and multicollinearity.

## Results

Of the 1559 patients admitted to the study wards during the study period, a total of 461 participants were recruited to this study, with mean age 76.2 ± 8.9 years, 56.8% female. The proportion of participants from each ward were 33.6% (cardiology), 23.2% (neurology), 18.7% (general medicine), 14.1% (endocrinology), and 10.4% (private general medicine ward). Data was not collected on those who were not recruited. The most prevalent comorbidities were hypertension, renal impairment, and stroke (Table 1). The prevalence of frailty was 31.9% according to the REFS. With Fried frailty criteria, the percentages of non-frail, pre-frail and frail participants were 24.5, 40.1 and 35.4%, respectively. In general, frail participants were older, had lower levels of education, poor reported nutritional status, underweight, and chronic renal impairment compared to the non-frail. In addition, compared to the robust, participants with a frailty status defined by the REFS had significantly higher proportions of females, living alone, and anaemia, while participants with a frailty status defined by Fried’s criteria had higher prevalence of cardiovascular diseases, especially heart failure and stroke.

**Table 1:** Participant general characteristics

Variables	All	Fried frailty phenotype			REFS		
		Non-frail (298)	Frail (163)	*P*	Non-frail (314)	Frail (147)	*p*
Age, years	76.2 ± 8.9	74.2 ± 8.5	79.8 ± 8.5	<0.001	74.7 ± 8.6	79.3 ± 8.7	<0.001
Female	262 (56.8%)	165 (55.4%)	97 (59.5%)	0.39	166 (52.9%)	96 (65.3%)	0.01
Level of education							
Not finished high school	286 (62.0%)	169 (56.7%)	117 (71.8%)	0.001	173 (55.1%)	113 (76.9%)	<0.001
Finished high school	88 (19.1%)	70 (23.5%)	18 (11.0%)		73 (23.2%)	15 (10.2%)	
Finished university	44 (9.5%)	34 (11.4%)	10 (6.1%)		38 (12.1%)	6 (4.1%)	
Higher education	43 (9.3%)	25 (8.4%)	18 (11.0%)		30 (9.6%)	13 (8.8%)	
Living alone	9 (2.0%)	6 (2.0%)	3 (1.8%)	0.90	3 (1.0%)	6 (4.1%)	0.02
Poor reported nutritional status	39 (8.5%)	14 (4.7%)	25 (15.3%)	<0.001	12 (3.8%)	27 (18.4%)	<0.001
BMI							
Underweight	114 (24.7%)	64 (21.5%)	50 (30.7%)	0.02	69 (22.1%)	45 (30.6%)	0.03
Normal	222 (48.2%)	146 (49.0%)	76 (46.6%)		156 (50.0%)	66 (44.2%)	
Overweight	104 (22.6%)	69 (23.2%)	35 (21.5%)		68 (21.8%)	35 (23.8%)	
Obese	21 (4.6%)	19 (6.4%)	2 (1.2%)		19 (6.1%)	2 (1.4%)	
Comorbidities							
Hypertension	288 (62.5%)	186 (62.4%)	102 (62.6%)	0.97	201 (64.0%)	87 (59.2%)	0.32
Renal impairment (GFR < 60 ml/min/1.73 m^2^)	240 (52.1%)	133 (62.4%)	107 (85.6%)	<0.001	156 (66.4%)	82 (81.2%)	0.01
Stroke	186 (40.3%)	111 (37.2%)	75 (46.0%)	0.07	130 (41.4%)	56 (38.1%)	0.50
Osteoarthritis	115 (24.9%)	67 (22.5%)	48 (29.4%)	0.09	75 (23.9%)	40 (27.2%)	0.24
Anaemia	115 (24.9%)	65 (29.3%)	50 (38.5%)	0.08	71 (29.0%)	44 (41.1%)	0.02
Diabetes	110 (23.9%)	69 (23.2%)	41 (25.2%)	0.63	72 (22.9%)	38 (25.9%)	0.49
Chronic pulmonary diseases	57 (12.4%)	34 (11.4%)	23 (14.1%)	0.41	38 (12.1%)	19 (13.0%)	0.78
Peripheral vascular disease/ aortic atherosclerosis	32 (6.9%)	20 (6.7%)	12 (7.4%)	0.80	26 (8.3%)	6 (4.1%)	0.10
Ischemic heart disease	29 (6.3%)	18 (6.1%)	11 (6.7%)	0.77	20 (6.4%)	9 (6.1%)	0.91
Heart failure	29 (6.3%)	13 (4.4%)	16 (9.8%)	0.02	19 (6.1%)	10 (6.8%)	0.76
Cancer	16 (3.5%)	8 (2.7%)	8 (4.9%)	0.21	9 (2.9%)	7 (4.8%)	0.30

Continuous data are presented as mean ± standard deviation. Categorical data are shown as n (%)

*BMI* body mass index, *GFR* glomerular filtration rate, *REFS* Reported Edmonton Frail Scale

Details of the individual components of each frailty definition were presented in Table 2. Notably, the prevalence of slow walking speed and reduced physical activity (as shown in the proportions of low physical activity component of the Fried phenotype and the reported physical performance component of the REFS) was high, with around half of the participants suffering from these conditions. Male participants had higher prevalence of weight loss, while female participants had higher prevalence of exhaustion, low physical activity, cognitive impairment, impairment in daily activities, poor self-description of health, and forgetting to take medications (Table 2).

**Table 2:** Components of Fried frailty phenotype and REFS

Components of Fried frailty phenotype and the REFS	*N* = 461	Male	Female	*P*
Components of Fried frailty phenotype:				
Weight loss	56 (12.1%)	31 (6.7%)	25 (5.4%)	0.03
Low grip strength	115 (24.9%)	47 (10.2%)	68 (14.7%)	0.32
Exhaustion	196 (42.5%)	72 (15.6%)	124 (26.9%)	0.01
Low walking speed	258 (56%)	111 (24.1%)	147 (31.9%)	0.51
Low physical activity	208 (45.1%)	82 (17.8%)	126 (27.3%)	0.04
Components of REFS:				
Cognition: clock drawing test				
No errors	226(49.0%)	127 (27.5%)	99 (21.5%)	<0.001
Minor spacing errors	91(19.7%)	32 (6.7%)	59 (13%)	<0.001
Other errors	144(31.2%)	40 (8.7%)	104 (22.5%)	<0.001
Health status				
Admissions to hospital in the past year				
No admission	188 (40.8%)	74 (16.1%)	114 (24.7%)	0.38
1–2 admissions	233(50.5%)	106 (23%)	127 (27.5%)	0.38
> 2 admissions	40 (8.7%)	19 (4.1%)	21 (4.6%)	0.18
Description of health				
Excellent/ very good/ good	39 (8.5%)	19 (4.1%)	20 (4.4%)	0.06
Fair	287 (62.3%)	133 (29%)	154 (33.3%)	0.06
Poor	135(29.3%)	47 (10.2%)	88 (19.1%)	0.03
Functional independence: activities requiring help				
0–1 activities	196 (42.5%)	96 (1.3%)	100 (41.2%)	0.09
2–4 activities	130 (28.2%)	51 (11.1%)	79 (17.1%)	0.09
5–8 activities	135 (29.3%)	52 (11.3%)	83 (18%)	0.04
Social support: someone able to help				
Always	430 (93.3%)	191 (41.4%)	239 (51.9%)	0.12
Sometimes	28 (6.1%)	7 (1.5%)	21 (4.6%)	0.11
Never	3 (0.7%)	1 (0.2%)	2 (0.5%)	0.06
Medication				
Using ≥5 medications	78 (16.9%)	32 (6.7%)	46 (10.2%)	0.39
Forget to take medication sometimes	129 (28.0%)	34 (7.4%)	95 (20.6%)	0.00
Nutrition: weight loss	56 (12.1%)	31 (6.7%)	25 (5.4%)	0.03
Mood*:* sadness or depression	114 (24.7%)	50 (10.8%)	64 (11.9%)	0.47
Incontinence	27 (5.9%)	11 (2.4%)	16 (3.5%)	0.48
Self-reported performance				
Can do heavy work around the house without help	218 (47.3%)	84 (18.2%)	134 (29.1%)	0.03
Can go up and down stairs without help	139 (30.2%)	57 (12.4%)	82 (17.8%)	0.30
Can walk 1 km without help	241 (52.3%)	88 (19.1%)	153 (33.2%)	0.01

Data are shown as n (%). *REFS* Reported Edmonton Frail Scale

Univariate and multivariate logistic regression of potential risk factors for frailty was presented in Table 3. In the final models, age, poor nutritional status and low education were associated with frailty defined by either Fried phenotype or REFS. The presence of cardiovascular diseases was associated with frailty defined by Fried phenotype (adjusted OR = 1.76, 95%CI 1.16–2.67) but not with frailty defined by REFS.

**Table 3:** Factors associated with frailty on univariate and multivariate logistic regression

Variables	Univariate		Multivariate	
	Odds ratio for frailty (95% CI)	*P*	Adjusted odds ratio for frailty (95% CI)	*P*
Frailty defined by Fried frailty phenotype				
Female gender	1.19 (0.80–1.75)	0.39	–	–
Age	1.08 (1.06–1.11)	<0.001	1.07 (1.05–1.10)	<0.001
Low education (not finish high school)	1.94 (1.29–2.93)	0.01	1.58 (1.01–2.46)	0.04
Poor reported nutritional status	3.68 (1.85–7.29)	<0.001	2.96 (1.43–6.11)	0.01
Cardiovascular diseases	1.57 (1.07–2.30)	0.02	1.76 (1.16–2.67)	0.01
Hypertension	1.01 (0.68–1.49)	0.97	–	–
Diabetes mellitus	1.16 (0.72–1.74)	0.63	–	–
Chronic pulmonary diseases	1.27 (0.72–2.24)	0.41	–	–
Renal impairment (GFR < 60 ml/min/1.73 m^2^)	3.58 (2.02–6.33)	<0.001	–	–
Cancer	1.87 (0.69–5.08)	0.22	–	–
Osteoarthritis	1.44 (0.93–2.22)	0.10	–	–
Anemia	1.51 (0.96–2.38)	0.08	–	–
Living alone	0.91 (0.23–3.70)	0.90	–	–
Frailty defined by REFS				
Female gender	1.68 (1.12–2.52)	0.01	–	–
Age	1.06 (1.04–1.09)	<0.001	1.05 (1.03–1.08)	<0.001
Low education (not finish high school)	2.71 (1.74–4.22)	<0.001	2.18 (1.37–3.46)	0.001
Poor reported nutritional status	5.66 (2.78–11.54)	<0.001	4.51 (2.15–9.44)	<0.001
Cardiovascular diseases	0.85 (0.57–1.26)	0.41	–	–
Hypertension	0.82 (0.55–1.22)	0.32	–	–
Diabetes mellitus	1.17 (0.75–1.84)	0.49	–	–
Chronic pulmonary diseases	1.09 (0.60–1.96)	0.78	–	–
Renal impairment (GFR < 60 ml/min/1.73 m^2^)	2.16 (1.22–3.81)	0.01	–	–
Cancer	1.69 (0.62–4.64)	0.31	–	–
Osteoarthritis	1.19 (0.76–1.86)	0.44	–	–
Anemia	1.71 (1.07–2.75)	0.03	–	–
Living alone	4.41 (1.09–17.89)	0.04	–	–

Only variables that had a P-value <0.20 in univariate regression were entered into multiple regression model

*REFS* Reported Edmonton Frail Scale, *GFR* glomerular filtration rate, *CI* confidence interval

## Discussion

In this study, we found that frailty was common amongst hospitalised older patients in Vietnam. The prevalence of frailty defined by Fried phenotype and REFS was rather similar, with 35.4 and 31.9%, respectively. This prevalence is consistent with studies about frailty in other low and middle income countries [13]. Previous studies showed that the prevalence of frailty in older people in developing countries is also quite high, from 5.4 to 44% in community-dwelling older adults, 27.8 to 71.3% in geriatric outpatients and 32.3 to 49.3% in institutionalised older patients, and Fried’s frailty phenotype was used to define frailty in the majority of studies [13]. The prevalence of reduced physical activity and slow walking speed was quite high amongst the participants of this study, consistent with a published study based on World Health Organisation’s SAGE data [25]. This evidence suggests a need for studies on frailty in developing countries as the long-term burden of chronic diseases is significant in the population living in these parts of the world [26, 27].

Besides chronological age, this study showed that a poor nutritional status and a low education were associated with frailty defined by either Fried phenotype or REFS in older patients in Vietnam. This is consistent with recent studies which have reported a significant association between frailty and poor nutritional status [28]. In a longitudinal study in older people in France, women who had energy intake less than 25 kcal/kg/day were more likely to become frail (with ten-year follow up) [29]. Inadequate protein intake may lead to a failure in maintaining muscle mass and function, and physical function [30]. Several observational studies suggested that protein supplementation may help decelerate frailty and the Asia-Pacific Clinical Practice Guidelines for the Management of Frailty also conditionally recommended caloric and protein supplementation in frail older people with weight loss [28, 31]. It is also evident that there is a strong correlation between frailty, fitness and economic indicators in older adults [32]. A study in Vietnam has shown that life expectancy has improved among those with better socioeconomic conditions and tends to decrease in the most vulnerable groups [33]. To our best knowledge, there have not been any published studies about nutrition in older people in Vietnam.

Interestingly, in this study cardiovascular disease was significantly associated with frailty defined by Fried phenotype. The relationship between frailty and cardiovascular disease has been established in many studies in older adults [34, 35]. This finding is meaningful as reports have shown that cardiovascular disease is the leading cause of death in Vietnam [36, 37]. Our current medical practices are disease-based and older patients with cardiovascular diseases are usually not assessed for frailty [38]. Frailty assessment could provide a window of opportunity to prevent adverse outcomes related to frailty in this population, such as falls and other geriatric syndromes.

To our best knowledge, this is the first study of frailty in older patients in Vietnam. The major limitation of this study is that patients admitted to the hospital during weekends and holidays were missed, and not all wards were included. Based on the hospital database, the total number of admissions to the targeted wards during the study period (from April 2015 to October 2015) was 1559. The recruiting team did not recruit participants during weekends, holidays and during the time that they were busy with other tasks such as examinations at schools and the duties at their workplaces. However, during the recruiting time, they made their best efforts to recruit consecutive patients. This study may also be prone to selection bias due to the unavailability of data of patients that were not screened for frailty. Participant cognitive screening was not conducted by the study team and participants’ cognitive status was identified based on their medical records. Another limitation of our study is that we just defined low physical activity based on participants’ report that “I rarely or never do any physical activities”.

## Conclusion

In conclusion, this study showed that frailty was common amongst older inpatients in Vietnam. Chronological age, poor nutritional status, low education and cardiovascular diseases were significantly associated with frailty in these patients. Further studies are needed to establish whether the associations between frailty and adverse outcomes observed in other ageing populations also apply to older Vietnamese inpatients. This has implications for health policy and planning for the ageing population in Vietnam. These findings also support further studies about nutrition in older people in Vietnam.

## Abbreviations

BMI: Body mass index
CES-D: Centre for Epidemiologic Studies Depression Scale
REFS: Reported edmonton frail scale
